# A multi-country One Health foodborne outbreak simulation exercise: cross-sectoral cooperation, data sharing and communication

**DOI:** 10.3389/fpubh.2023.1121522

**Published:** 2023-06-13

**Authors:** Frederico Alves, Karin Artursson, Juliette Bloch, Anne Brisabois, Hein Imberechts, Pikka Jokelainen, Roberto La Ragione, Mats Lindblad, Rebecca Litzell Forss, Denise A. Marston, Omid Parvizi, Lena Tuominen, Anna Omazic

**Affiliations:** ^1^Department of Infectious Diseases, National Institute of Health Dr. Ricardo Jorge (INSA), Lisbon, Portugal; ^2^Office of Science and International Collaboration, National Veterinary Institute (SVA), Uppsala, Sweden; ^3^Department of Health Alerts and Vigilances, French Agency for Food, Environmental and Occupational Health & Safety (ANSES), Maisons-Alfort, France; ^4^Department of Strategy and Program, French Agency for Food, Environmental and Occupational Health and Safety (ANSES), Maisons-Alfort, France; ^5^Sciensano, The Belgian Institute for Health, Brussels, Belgium; ^6^Infectious Disease Preparedness, Statens Serum Institut, Copenhagen, Denmark; ^7^School of Veterinary Medicine, University of Surrey, Guildford, Surrey, United Kingdom; ^8^School of Biosciences, University of Surrey, Guildford, Surrey, United Kingdom; ^9^Department of Safe Food, Swedish Food Agency, Uppsala, Sweden; ^10^Institute of Bacterial Infections and Zoonoses, Friedrich-Loeffler-Institut (Federal Research Institute for Animal Health), Jena, Germany; ^11^Department of Chemistry, Environment and Feed Hygiene, National Veterinary Institute (SVA), Uppsala, Sweden

**Keywords:** *Salmonella*, simulation exercise, zoonosis, One Health, foodborne outbreak, public health, food safety, animal health

## Abstract

**Introduction:**

The awareness of scientists and policy makers regarding the requirement for an integrated One Health (OH) approach in responding to zoonoses has increased in recent years. However, there remains an overall inertia in relation to the implementation of practical cross-sector collaborations. Foodborne outbreaks of zoonotic diseases continue to affect the European population despite stringent regulations, evidencing the requirement for better ‘prevent, detect and response’ strategies. Response exercises play an essential role in the improvement of crisis management plans, providing the opportunity to test practical intervention methodologies in a controlled environment.

**Methods:**

The One Health European Joint Programme simulation exercise (OHEJP SimEx) aimed at practicing the OH capacity and interoperability across public health, animal health and food safety sectors in a challenging outbreak scenario. The OHEJP SimEx was delivered through a sequence of scripts covering the different stages of a *Salmonella* outbreak investigation at a national level, involving both the human food chain and the raw pet feed industry.

**Results:**

A total of 255 participants from 11 European countries (Belgium, Denmark, Estonia, Finland, France, Italy, Norway, Poland, Portugal, Sweden, the Netherlands) took part in national level two-day exercises during 2022. National evaluations identified common recommendations to countries aiming to improve their OH structure to establish formal communication channels between sectors, implement a common data sharing platform, harmonize laboratory procedures, and reinforce inter-laboratory networks within countries. The large proportion of participants (94%) indicated significant interest in pursuing a OH approach and desire to work more closely with other sectors.

**Discussion:**

The OHEJP SimEx outcomes will assist policy makers in implementing a harmonized approach to cross-sector health-related topics, by highlighting the benefits of cooperation, identifying gaps in the current strategies and suggesting actions required to better address foodborne outbreaks. Furthermore, we summarize recommendations for future OH simulation exercises, which are essential to continually test, challenge and improve national OH strategies.

## 1. Introduction

Detecting and responding to current and emerging zoonotic threats increasingly requires involvement from more than just one sector. Therefore, fostering cross-sector collaboration and disease response preparedness under the framework of One Health (OH) has become a priority for many countries (1). The awareness of the scientific community and policy makers to the emerging risk that infectious pathogens pose to health has increased due to the efforts made in the OH field, with multiple international projects showing the way to further developing this area (2). OH is defined as “an integrative and systemic approach to health, grounded on the understanding that human health is closely linked to the healthiness of food, animals and the environment, and the healthy balance of their impact on the ecosystems they share, everywhere in the world” (3). Several international reports reveal a general agreement among stakeholders regarding the benefits that a One Health approach brings to society, contributing to tackle food and water insecurity and shortage, supporting a sustainable development, and helping in the management of financial and natural resources toward future risk prevention (4–7).

While the theoretical aspects of OH have been well established and embraced within the scientific community, practical implementation has been hindered due to the complex requirement of political, ethical, economical, and societal engagement (8), rendering a truly unified and efficient One Health based system far from being delivered. Initiatives which aim to achieve a tangible transformation should primarily focus on improving communication, coordination, collaboration, and capacity building across all sectors of society and to align with the fundamental principles of inclusivity, parity and stewardship (9). The One Health European Joint Programme (One Health EJP)^1^ was conceived to move toward a holistic global approach to health threats, with the primary goal of promoting international and interinstitutional collaboration to improve preparedness. The One Health EJP consortium promotes transdisciplinary collaboration across sectors by supporting collective research activities and developing tools and guidelines in the fields of foodborne zoonoses, antimicrobial resistance, and emerging threats. In addition, by providing education and training initiatives, the consortium facilitates the harmonization of the approaches taken by different institutes. Congregating 44 partners across Europe, building upon the collaborations from the Med-Vet-Net-Association^2^, the One Health EJP is based on the concept that no transmissible disease can be addressed as a problem constricted to any individual country or sector (10). The consortium strives to employ the outputs delivered and promote them across the scientific community, thereby implementing tangible changes that can be sustained beyond the duration of the programme.

Food safety and security are considered an overarching subject in the OH international agenda for a roadmap toward sustainable development (2, 11, 12). Despite the rigorous regulation enforced within the European Union (EU), foodborne outbreaks continue to significantly affect the population with a sustained number of reported outbreaks each year (13). This impact showcases the importance of equipping response systems with improved tools to address and mitigate foodborne infections. For example, despite the strictly regulated control programmes implemented in poultry production units and the regulation on food safety and process hygiene criteria for *Salmonella enterica* serotype Typhimurium in several food categories, it remains as an important gastrointestinal pathogen in EU, accountable for 22% of all foodborne outbreaks in 2020 (13). Despite egg and egg products being the most common sources of *Salmonella,* other foodstuffs such as meat products also contribute to human infections (14), highlighting the need to identify additional infection routes (15). Only by linking together the sector specific activities, thereby embracing the ethos of the OH approach, will we improve our response to less predictable outbreaks of disease.

While disease incursions remain a constant and significant threat, our ability to adequately respond to them defines their scale and impact on the community. An essential tool within emergency preparedness plans is the conduction of simulation exercises, exposing existing gaps in a controlled environment and assessing the current crisis management strategies without the negative consequences of a real-life emergency. Improvement plans drawn up after such an exercise provides detailed and tangible documentation for each sector and motivation to deliver the improvements required. The nature and scale of the exercise may vary depending on the aims and objectives, budget, and resource availability, ranging from discussion-based exercises (orientation exercise; table-top exercise) to more complex operation-based exercises (drill; functional or command post exercise; full-scale exercise). Table-top exercises are a common format for simulation exercises, offering the opportunity to be completed in an informal and stress-free environment where the participants are guided by a facilitator and encouraged to engage in a roundtable discussion based on a simulated scenario. A series of scripted injects are given to the participants, presenting the problems that need to be tackled. This type of exercise stimulates the participants’ problem-solving capacities and develops the communication strategies required to respond effectively in the event of an actual disease incursion. Although table-top exercises lack the full realism of functional or full-scale exercises, they provide an effective and efficient way to become familiar with procedures and policy. Moreover, this format is not necessarily timebound, therefore allowing the participants to allocate time to focus on the critical elements of the scenario (16–18).

Within the remit of the One Health EJP, the multi-country OHEJP SimEx was designed with an overall aim to practice the OH capability, capacity, and interoperability at a national level, across public health (PH), animal health (AH) and food safety (FS) sectors. To succeed in this aim, a challenging outbreak scenario with a zoonotic disease that typifies the OH concept and that was relevant across Europe was required. Therefore, a *Salmonella* outbreak scenario was developed, which included both human food and pet feed supplies specifically to provide the opportunity to share experiences and perspectives across sectors, evidencing the added value of applying a OH approach, while also providing an opportunity to test a food tracing software tool: The FoodChain-Lab (FCL) web application. By assisting countries to identify current gaps in their OH approach to a foodborne outbreak and defining strategies to tackle them, the OHEJP SimEx outcomes have resulted in recommendations suitable for all countries to assist in defining a national roadmap for future outbreak preparedness plans.

## 2. Methods

### 2.1. Organization and planning

**Figure 1:**
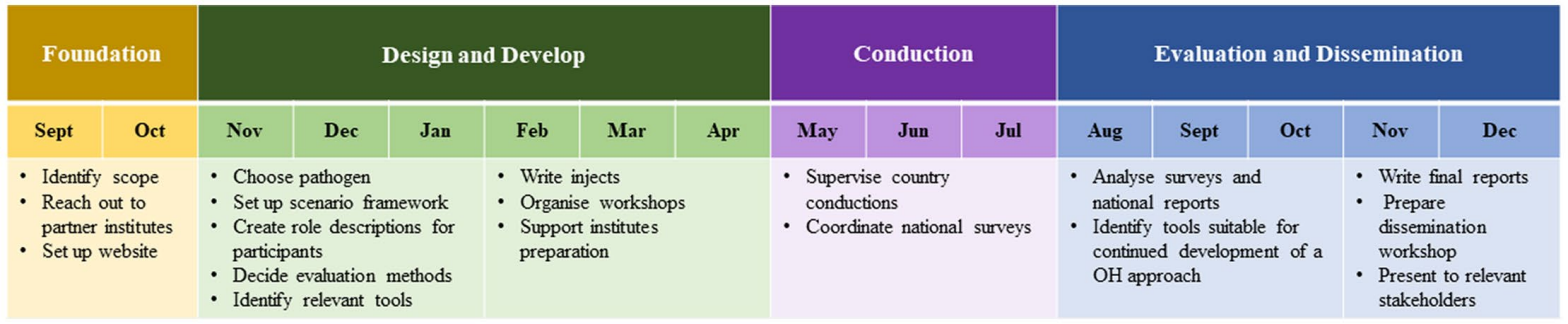
Timeline of the OHEJP SimEx project planning, conducting, evaluation and dissemination activities. The project began in September 2021 and ended in December 2022.

Within the One Health EJP, a Joint Integrative Project (JIP) priority topic was identified: “Sharing best intervention practice – twinning and simulation exercises.” To address this topic, the OHEJP SimEx project was designed. A OHEJP SimEx Steering Board was formed with representatives from the One Health EJP Project Management Team. The Steering Board provided the Project Directive. Relevant stakeholders, European Centre for Disease Prevention and Control (ECDC), European Food Safety Authority (EFSA), Food and Agriculture Organization of the United Nations (FAO), World Organisation for Animal Health (WOAH) and World Health Organization (WHO) were represented in an Advisory Board. The timeline for the project was constrained within the overall OHEJP project timeframe. Preparation for the project began in January 2021 with the appointment of a project leader and included recruitment of an international project team of nine specialists with complementary expertise in the areas of PH, AH and FS and emergency response exercises. The team was responsible for planning, supporting the national conductions, evaluating the outcome of OHEJP SimEx and dissemination of the outcomes which began in September 2021 and completed in December 2022 (Figure 1). Meetings between the project team and the Steering Board were held on a regular basis throughout this period to ensure the scenario and outputs remained relevant and applicable to the overarching One Health EJP aims. Dissemination of the project outcomes continued after the project completed through the continuing communication channels within One Health EJP.

The OHEJP SimEx project team developed a realistic scenario which could be executed in multiple European countries including the following criteria:

1. The pathogen must be relevant across Europe
2. The pathogen must satisfy the One-Health focus spanning AH, PH and FS
3. Country level focus
4. Cross sectoral collaboration focus
5. Data sharing focus

This exercise was included as an implementation activity in One Health EJP. All One Health EJP partner institutes were invited to participate in the OHEJP SimEx. In addition, the institutes were encouraged to invite other institutes from outside the One Health EJP consortium, e.g., to cover up for missing sectors or to better represent the national outbreak management. Participation of institutes was on a voluntary basis. The original request and subsequent reminders to participate in the exercise were sent out by email to the following groups within the One Health EJP: Scientific Steering Board members, representatives from the Programme Managers’ Committee and Project Leaders. All partners that decided to participate selected a contact person who become the link between the institute and the One Health SimEx Project Team. The contact person from each institute was then fully involved in the decision-making process about participation. In November 2021, 15 countries had expressed their willingness to participate in the exercise. By the time of the preparatory workshop (see Section 2.3) for the National Exercise Leaders (NELs) and Local Exercise Leaders (LELs) in March 2022, two countries had decided to withdraw due to their national outbreak teams being heavily involved in the COVID-19 pandemic and avian influenza outbreak responses. A further two countries withdrew due to difficulties in involving all sectors and changes in leadership, respectively. Thus, conduction of the scenario involved eleven countries (Belgium, Denmark, Estonia, Finland, France, Italy, the Netherlands, Norway, Poland, Portugal, and Sweden).

**Figure 2:**
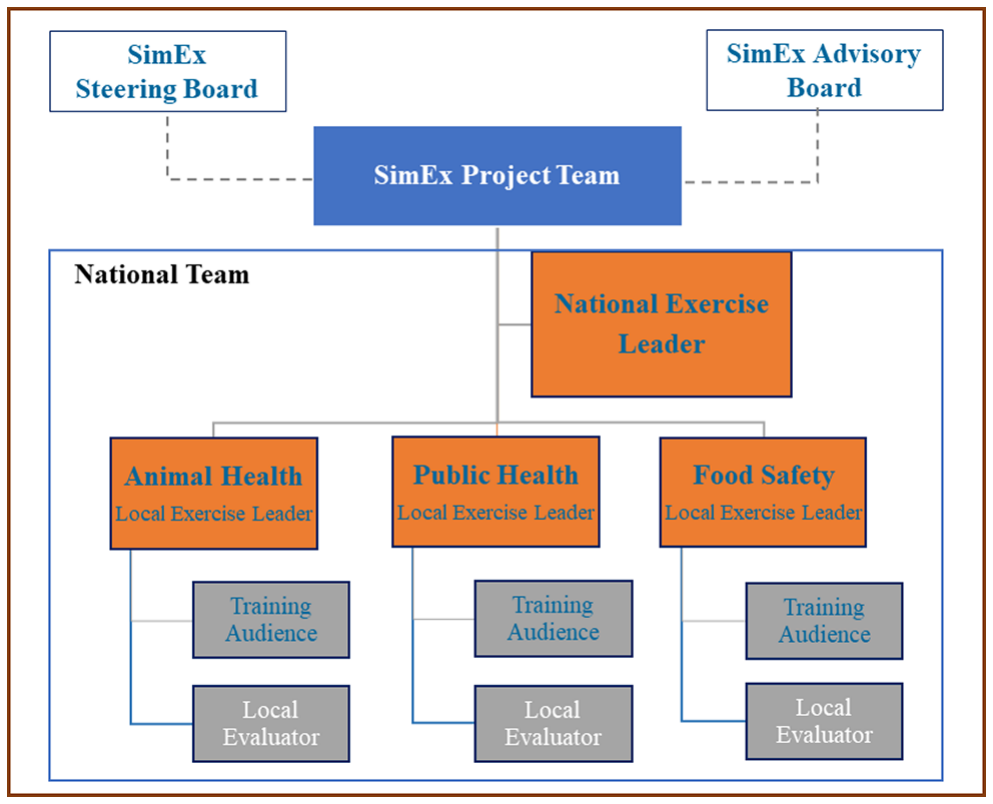
Organogram of the OHEJP SimEx project. The SimEx team was supported by both the Advisory board (including experts in outbreak exercises) and the Steering board. Each national team (denoted by the blue box) is led by the National Exercise Lead (NEL), coordinating the sector Local Exercise Leads (LEL) who in most cases represent a specific Institute. The Local Evaluators (LEs) were chosen based on their specialist knowledge in each sector, and the Training Audience (TA) chosen by the LELs.

Then, in order to conduct the exercise at national level, it was necessary for each participating country to assemble a national team, including representatives from PH, AH and FS. Each national team was composed of a NEL, LELs, Local Evaluators (LEs), and a Training Audience (TA) (Figure 2). Each participating institute appointed a LEL, whose role was to organize and facilitate the institute’s participation in the initiative by establishing a connection between the OHEJP SimEx project team and the institute, identifying and convening a TA, and guiding the country conduction. The NEL had overall responsibility for the coordination of the team at country-level and for most countries the NEL was also one of the LELs. The NELs and LELs had the option to adapt the scenario to the relevant local setting and to add further institutional and national objectives to the OHEJP SimEx. The NEL and LELs assembled the TA ensuring inclusion from each sector and varying levels of experience and seniority. Typically, the TA included epidemiologists, medical experts, veterinarians, laboratory personnel, communication experts and other representatives from the relevant sectors. Each institute appointed a LE, responsible for the evaluation of the exercise both during and after conduction. The LEs were critical in the success of the project, providing key observations that identified the existing gaps hindering a true One Health cooperation.

The OHEJP SimEx was designed as a table-top exercise in which the participants were encouraged to meet in person for the conduction. Final decisions regarding organization of the conduction were made by the national teams. The OHEJP SimEx ran for 2 days and was conducted in the participating countries during the period of May to September 2022.

### 2.2. Scenario, objectives and conduction

The OHEJP SimEx was built following the ECDC guidelines on simulation exercises (18). The exercise was designed to replicate a *Salmonella enterica* serotype Typhimurium outbreak at a national level involving both the human food chain and the raw pet feed industry. To ensure that a cohesive language was used between sectors, the OH glossary produced within the One Health EJP was used (19, 20). The criteria listed in 2.1 were defined at the beginning of the project to guide the OHEJP SimEx project team in the scenario design with the purpose to help participating countries to identify gaps in their national outbreak contingency plans. The scenario covered all stages of a foodborne outbreak investigation and considered different possible routes of transmission between humans and animals. As the scenario unfolded, the TA was challenged with a sequence of injects covering relevant outbreak related information (i.e., number of cases, epidemiological data, laboratory results). Each inject was designed to trigger discussion and encourage the sectors to work together, showcasing the added value of employing a OH approach in a zoonotic outbreak situation.

The finalized exercise scenario was delivered through a sequence of 15 scripted injects divided into three parts. The first part of the exercise focused on increasing knowledge with objectives that highlighted the role and functionality of each sector and the availability of guidelines and systems in the event of a zoonotic outbreak. The second part of the exercise was designed to emphasize the importance of data sharing in an outbreak situation and help national teams identify possible gaps in the cohesiveness of current data collection practices. The final part of the exercise was designed to promote intersectoral cooperation and communication in an outbreak situation, helping the TA improve their understanding on how to create common main messages and identify relevant target audiences.

Each inject consisted of two parts, one to be delivered exclusively to the LELs covering the purpose of the inject, the expected outcomes, critical conditions for TA to achieve in order to proceed, and some follow up questions. The other part was for the TA with the event to be worked on. While most injects targeted the whole TA, some were directed toward a specific sector, to mimic a real-life situation and assess the flow of information between the sectors. The exercise scenario is available from the corresponding author upon request.

Prior to conduction, all NEL and LELs attended a workshop held by the OHEJP SimEx project team, during which the scenario was presented NELs and LELs were encouraged to review and adapt the scenario to reflect their national setting, if necessary. Providing the flexibility to tailor the scenario allowed the NEL and LELs to ensure maximum relevance for the training audience.

FoodChain-Lab web application is a food tracing software jointly developed by the German Federal Institute for Risk Assessment (BfR), EFSA, One Health EJP COHESIVE and other European projects, which allows to model, visualize, and analyze complex food supply chain networks (21, 22). This tool was included in the OHEJP SimEx as a practical tracing exercise, for the TA to establish possible contamination sources and transmission chains. Inclusion of FCL which could be accessed by all sectors highlighted the advantages of having an intersectoral tool when deciding on the implementation of control measures like product sampling and batch recall.

A final meeting at the end of the exercise allowed the TA to review and discuss the challenges encountered during the simulated outbreak investigation and management.

### 2.3. Evaluation of outcomes

The OHJEP SimEx evaluation was designed to assess the success of the objectives to identify any limitations on data sharing, develop intersectoral communication strategies and increase the mutual understanding between sectors. By identifying cooperation gaps, the evaluation also provided evidence to support the improvement of future foodborne outbreak management strategies with a OH approach.

The LEs attended a training session, delivered by the OHEJP SimEx project team prior to conduction to prepare and support them. This included how to conduct After-Action Review (AAR; Hot debrief). The guided AAR discussions covered the chronological narrative of the conduction, focusing on the most relevant decisions to highlight the strengths and weaknesses identified. Hot debriefs provided participants with the vital opportunity to share their thoughts while the experience is still fresh, avoiding missing relevant details. Post-conduction, a link to a survey was sent to all participants (i.e TA, NELs, LELs and LEs), to provide the project team with invaluable feedback on their experiences. To guarantee representative value, a minimum response rate of 80% was aimed for. The majority of questions were posed according to the Likert scale with four different options: strongly disagree, disagree, agree, strongly agree. To facilitate the interpretation of the feedback, these options were reduced into two categories: disagree and agree. Answers were processed in Microsoft Excel (version 2210 Build 16. 0. 15726. 2018816.43; Microsoft, Washington, USA) and presented as descriptive statistics.

The LEL of each institute was responsible for analyzing its own outcomes which were combined by the NEL to deliver a national report covering the experiences of the conduction, main lessons learned and recommendations for future improvement. The OHEJP SimEx project team provided a template for the national report to ensure a level of consistency in the information provided. The OHEJP SimEx project team analyzed the national reports to identify common problems, major gaps, and current best practices. However, because the report template did not explicitly request answers to a series of questions, the data presented below was compiled from the information provided and may not represent a complete picture.

By compiling and analyzing data from all the evaluation outcomes, we have provided a comprehensive analysis and summarized a list of recommendations for the improvement of OH approach to foodborne outbreaks as well as suggestions for future OH simulation exercises. In addition, the national experiences were shared at an internal One Health EJP Scientific Steering Board meeting (28th of September 2022) and at a dedicated Joint SimEx/Dissemination Workshop ‘A One Health simulation exercise as a roadmap for future foodborne outbreak preparedness’ (6th December 2022) that was targeted to stakeholders.

### 2.4. Ethical statement

This research was conducted in accordance with the principles embodied in the Declaration of Helsinki and in accordance with the One Health EJP Consortium agreement, project number 773830, Version 4, 2017-12-13 (signed version) with Amendment #1–2020. This consortium agreement is based upon regulation (EU) No 1290/2013 of the European Parliament and of the Council of 11 December 2013 laying down the rules for the participation and dissemination in “Horizon 2020 – the Framework Programme for Research and Innovation (2014–2020).” The data in the post-exercise survey, completed by the participants, were collected *via* an electronic questionnaire in EUSurvey (23) set in anonymous mode, and no personal data were collected. Participant were informed at the start of the survey that the results would be collated and published publicly. Individual written informed consent was not required from the participants.

## 3. Results

**Figure 3:**
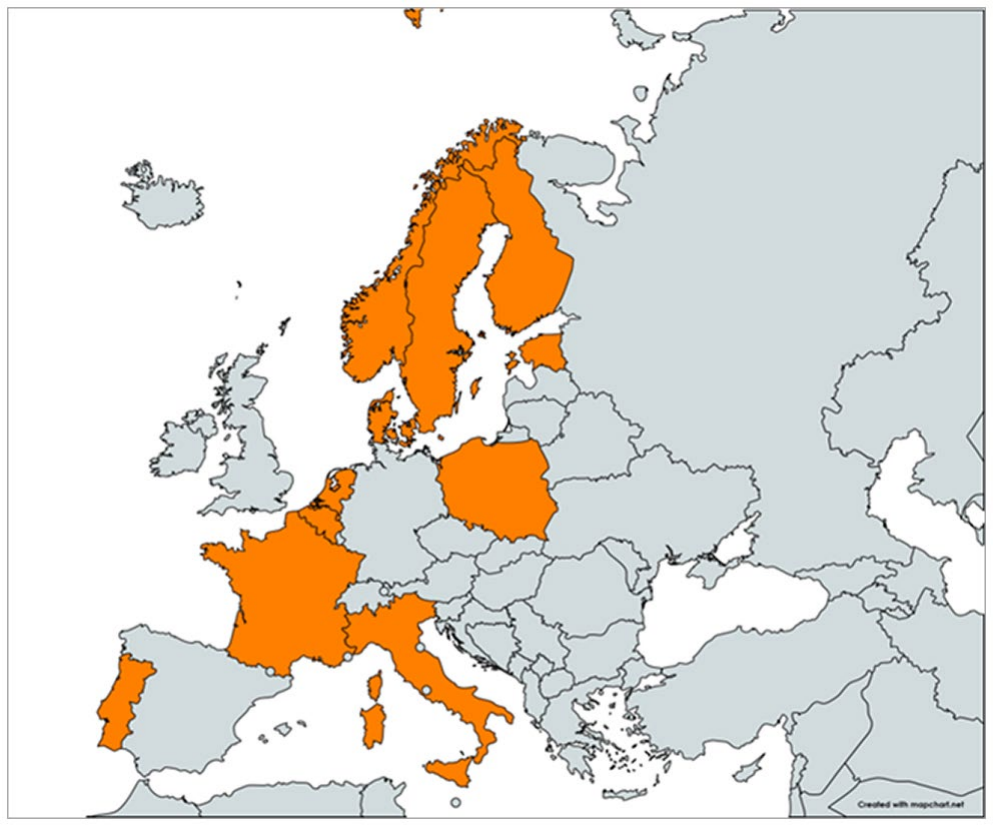
Map of the participant countries. Participating countries are highlighted in orange.

In total, 255 participants from 42 institutions from 11 countries (Belgium, Denmark, Estonia, Finland, France, Italy, Norway, Poland, Portugal, Sweden, the Netherlands) completed the OHEJP SimEx (Table 1; Figure 3), from which 205 answered the post-conduction survey. Four countries achieved the desired 80% response rate, and all countries had response rates above 60%. The overall response rate was 80% (*n* = 205), confirming that the results can be considered representative.

**Table 1:** Number of participants and post-conduction survey response rate of national teams.

Country	Number of participants	Post-conduction survey respondents	Response rate (%)
Belgium	37	29	78.4
Denmark	23	19	82.6
Estonia	20	15	75.0
Finland	19	13	68.4
France	25	16	64.0
Italy	52	45	86.5
Norway	23	18	78.3
Poland	13	10	76.9
Portugal	11	8	72.7
Sweden	21	21	100
The Netherlands	11	11	100
Total	255	205	80.4

Based on the post-conduction survey results, there was a balanced representation across the three sectors, with 23% (*n* = 47) of participants belonging to the AH sector, 35% (*n* = 71) to the FS sector and 37% (*n* = 75) to the PH sector. Twelve participants (6%) did not identify with a sector. The overall opinion on the exercise was positive, with 94% (*n* = 192) of participants reporting feeling encouraged to pursue a OH approach by working more closely with other sectors in future outbreak situations.

### 3.1. Exercise planning and conduction

The majority of the participating countries decided that the scenario was suitable to utilize as provided. However, minor adaptations were made by four out of the 11 countries. Modifications included adding information to explore topics of national relevance or providing supporting documents to the TA. In addition, one country made more significant changes to the scenario to replicate the structure of their national system. Four countries chose to translate the documents into a local language prior to conduction. Over 95% of participants reported finding the scenario (*n* = 195) and chosen pathogen (*n* = 201) relevant, 89% (*n* = 183) expressed that the scenario equally covered all the sectors, and 95% (*n* = 194) considered the scenario was in line with the exercise objectives. Sixteen percent (*n* = 32) of participants reported a lack of reality in the way the outbreak unfolded and 26% (*n* = 54) of participants did not think the injects fully mimicked a real-life outbreak situation.

At a country level, the TA size ranged from 5 to 40 individuals, with a median of 11. One challenge reported during the planning phase was the assembling of a TA with sufficient expertise to conduct a fruitful discussion while balancing the inclusion of less experienced staff that could benefit from this training opportunity. Two countries were not able to assemble representatives from all the relevant sectors which likely reflected some of the TA responses regarding their satisfaction of the exercise.

Organization of the facilities during conduction varied amongst the participating countries. Using one large room and seating the TA according to sector was reported as beneficial by resembling the reality of interinstitutional collaboration. The majority of countries either used a single large table or separated the TA around smaller tables by sector whilst ensuring intersectoral communication was still possible. The LEs mostly assumed a position separated from the TA. Five countries opted to conduct the OHEJP SimEx online. The importance of having a cohesive TA from the beginning to the end was evidenced by the problems reported by countries (*n* = 2) that experienced changes in the TA members during the exercise conduction, hindering continuity from 1 day to the next.

The post-conduction survey results noted positive feedback on the exercise organization, with over 94% of all participants either agreeing or strongly agreeing with aspects related with the time (*n* = 197) and venue (*n* = 191) logistics and 98% (*n* = 201) expressing their satisfaction with the performance of the NELs and LELs. The time frame for the sequence of events and the discussion time allocated to each inject worked well for the majority of countries, of those that did not agree included that the time frame did not resemble the country’s reality and that not enough time was allowed for discussion.

Holding preparation and planning meetings prior to the conduction was considered a benefit by the LEL and NELs for a successful conduction and was translated to a higher understanding amongst the TA of their role in the exercise and on the expected outcomes. Dividing the responsibilities of conduction between the LELs, depending on their expertise, was considered advantageous, as it reinforced the sense of a shared responsibility among different sectors.

Inclusion of FCL in the exercise was considered an opportunity for participants from AH and PH to better understand FS tracing procedures. The overall opinion on FCL varied, with most, 93% (*n* = 191), considering it useful for the exercise and some even requesting a more extensive practical exercise. Participants not directly involved in outbreak investigations and tracing, e.g., the communication experts, were less integrated in this part of the exercise.

Inevitably the multi-country approach revealed differences in perception of the scenario between national TAs. While one country reported the scenario as not challenging enough, another country deemed it unrealistic.

### 3.2. Scenario part 1: roles and functionality

**Figure 4:**
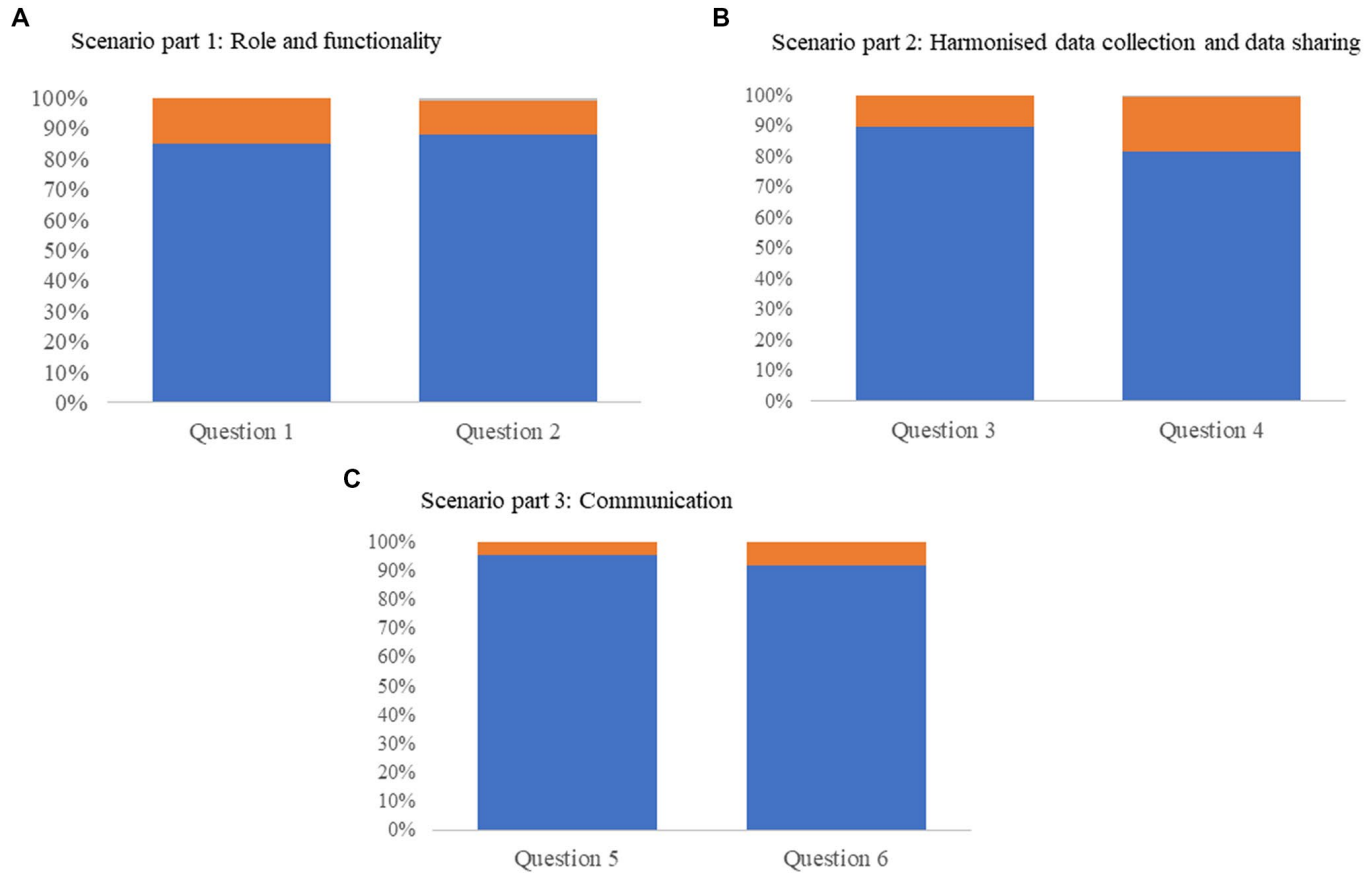
Post-conduction survey results graphically represented as percentage of respondents where blue indicates the respondent agrees, orange indicates the respondent disagrees, and gray indicates data missing. **(A)** Data from questions relating to scenario part 1: Role and functionality. Question 1: ‘This exercise has helped you to be more aware of the currently available warning systems and emergency action plans in place (both at national level and in the European Union) and when they should be activated,’ question 2: ‘Your understanding of what other sectors expect from your sector has increased.’ **(B)** Scenario part 2: Harmonized data collection and data sharing. Question 3: ‘You have gained an increased understanding of the need to have a harmonized approach for data collation when dealing with a foodborne zoonosis outbreak’, question 4: ‘Solving problems associated with data sharing is something your institute prioritizes.’ **(C)** Scenario part 3: Communication. Question 5: ‘The exercise clarified the importance of having a coordinated action plan,’ and question 6: ‘You gained a better understanding of the different communicational needs and different target audiences.’

The overall opinion among the participants, 88% (*n* = 180), stated the exercise was successful in highlighting the role of each sector in an event of a foodborne outbreak, and in showcasing the functionality of the systems in place (85% (*n* = 174) agreement) (Figure 4). Five countries also identified OHEJP SimEx as an opportunity for young professionals to familiarize themselves with the standard operating procedures and institutional routines to be followed during an outbreak. The exercise acted as a knowledge transfer platform between the less experienced and more experienced participants. OHEJP SimEx also provided institutes with the opportunity to revise their internal coordination practices including collaboration between different structural units of the same institute.

Three countries noted that an outbreak management team is only assembled once an outbreak has been declared, resulting in a fragmented decision-making process in the absence of a cohesive multidisciplinary team. One country reported that they have a long-standing collaboration for outbreak investigations and management.

Several countries highlighted the role of OHEJP SimEx in bringing people together and helping to strengthen interpersonal relations between professionals across sectors. In particular in the countries where the sector organization was more dispersed, OHEJP SimEx provided a unique opportunity for people to meet and clarify their roles. Moving toward or strengthening a single cooperating food safety governance structure, including both the human food chain and animal feed seemed to be the preferred system.

The need for further training initiatives covering institutes at different hierarchical levels to promote a common understanding between all parties involved and a quicker implementation of the necessary legal actions (e.g., product recall and inspections) was highlighted in several country reports. Four countries mentioned the key role of National Reference Laboratories in bridging the gaps between the different sectors and authorities, revealing the advantages of including them in the training initiatives.

One gap highlighted by four countries was that most interinstitutional communication was based on personal contacts and established through informal communication routes. The advantages of contacting professionals from other sectors using a personal contact network are largely recognized as a quick and efficient communication method, but a dependence on private networks is vulnerable particularly when there is a change in personnel.

Although not specifically focused on as it is outside the remit of a national response, it was evident that most countries had excellent knowledge and functionality of international early warning systems, e.g., the EpiPulse ECDC tool (24), which appeared well implemented in most countries. However, lack of full understanding of the available tools at national level and on how and when to activate them was evident.

### 3.3. Scenario part 2: harmonized data collection and data sharing

The second part of the scenario focused on harmonized approaches to data collection. The results showed that the OHEJP SimEx allowed different sectors to explore their data sharing procedures and to identify possible gaps that may hinder a coordinated and common data management plan. While 90% (*n* = 184) of the participants reported to have improved their understanding of the importance of data harmonization practices after conduction, 18% (*n* = 36) indicated that their institute did not prioritize the implementation of such practices (Figure 4). Furthermore, it was interesting to note that the degree of implementation of data sharing routines prior to the exercise conduction varied greatly amongst the participating countries. The majority of the participating countries (73%, *n* = 8) reported a requirement for an interinstitutional data sharing and data collection platform accessible to all sectors. Fragmented data collection structures, designed and implemented at an institute level, were considered to result in incompatible outputs and/or restrictive sharing policies, and proved inadequate for the OH scenario explored in this exercise.

Further investigation revealed that with the systems available in most countries, a sector is only contacted by other sectors at the point in which it becomes directly involved in an outbreak. This ‘need to know’ approach results in early-stage information being excluded from certain sectors and promotes inconsistency in accessible data to the investigation teams, reinforcing their dependence on informal data sharing routes to gain a OH perspective. In particular, data system gaps were evidenced in countries where the official control plans for raw pet feed are currently under development as pet raw food feeding is not widely practiced. Extrapolating these results to an emerging zoonotic disease scenario or an outbreak with an obscure source of infection, the benefits of a cross-sectoral surveillance network will become markedly evident, allowing for a more efficient, rapid, and adaptable response system.

One of the major challenges identified was how to comply with the General Data Protection Regulation (GDPR) that applies to different data. The participants raised legal questions on data accessibility, under which circumstances they can be accessed, and for what they can be used. Most participants also noted a need for clearer guidelines on GDPR in relation to pathogen isolates and microbial genomic information. Other challenges identified by the participants included the lack of political will, the absence of harmonized data collection methodologies and the need for further training in data analysis, particularly in the area of whole genome sequencing (WGS).

### 3.4. Scenario part 3: communication

Three countries expressed the importance of an early communication strategy during an outbreak investigation, and one country also stated that it is important to ensure a unified message across all sectors. To achieve this, it is necessary to involve communication experts from an early stage of the outbreak investigation and to clarify the role of each authority. Indeed, three countries mentioned that good communication between authorities has been previously established by holding regular joint meetings. Among participants, 92% (*n* = 189) expressed having increased their understanding of communicational needs and target audience identification (Figure 4).

Communication at an early stage of an outbreak can be challenging particularly when there is limited information available. One country discussed the effect of circulating misinformation to their public messaging strategy.

Through the scenario, the countries recognised successful communication occurs when the message clearly indicates the known, acknowledges what is still unknown, and indicates what is being carried out to acquire further information. This format reassures the public that the authorities are working in accordance with their duties and helps to reduce public concerns. In addition to this simple communication formula, uncertainties should also be communicated appropriately. Clear and transparent communication is expected to support and maintain trust in the authorities.

Variation in the perception of ‘severity’ between the different sectors during the early stages of outbreak was highlighted by one country. Concerns about the possibility of conflicting opinions arising between sectors and also between the outbreak investigation team and the communication experts were discussed. Indeed, a different country reported friction regarding whether to hold a press conference or not. Another country’s communication team also expressed concern that it may be unclear to the public which authority has primary responsibility for the outbreak investigation. The major gaps relating to risk communication were associated with a lack of structure for supporting communication strategies. Improving communication was highlighted by one country as the main action needed to further improve cross-sector cooperation.

### 3.5. Recommendations for One Health improvement

Regardless of the level of OH experience and maturity level of OH structures in the participating countries, there was an overall agreement on the major gaps and needs for improvement amongst the participants and countries (Table 2). These conclusions can be used by decision makers when reviewing the outbreak investigation and management plans in place at national or regional level and to define strategies to improve them. Furthermore, those planning future simulation exercises, wishing to integrate One Health coordination when responding to a health crisis, can benefit from the leant experiences from this exercise by considering the recommendations identified (Table 3).

**Table 2:** Recommendations for the improvement of the One Health approach to foodborne outbreaks.

Focus	Recommendation
Role and functionality	Create One Health strategies, guidelines and procedures at institutional level
	Hold regular meetings and training with authorities from all sectors
	Improve coordination between regional and central authorities
	Implement official communication channels between institutes
Harmonized data collection and data sharing	Harmonize typing methods and reinforce inter-laboratory networks
	Strengthen the links between human and veterinary primary health care practitioners and the official laboratories
	Implement common data collection and data sharing platforms that can be used across sectors
	Provide training in genomic data analysis and interpretation
Communication	Create a communication plan for outbreak situations

**Table 3:** Recommendations for future One Health simulation exercises.

Focus	Recommendations
Exercise Logistics	Hold preparation and planning meetings prior to conduction
	Divide the responsibilities of conduction between the facilitators from different sectors
	Incorporate practical tasks in the exercise
One Health	For a more detailed multi-country exercise, identify countries with similar systems for the scenario chosen for analysis
	Consider including more sectors (e.g., environmental sector)
	Have stronger focus on communication strategies
	Consider evaluating One Health-ness before and after the exercise

## 4. Discussion

Well-functioning preparedness plans for responding to unexpected events are a high priority for many countries due to increased health threats posed by climate change and globalization. As part of a broader contingency, conducting exercises should be considered a fundamental element, together with allocating resources, investing in equipment, and drawing action plans. Training initiatives such as OHEJP SimEx play an essential role in the national contingency, bringing relevant professionals from different sectors with appropriate expertise albeit with different level of experience together to promote a cohesive approach to future health emergency situations (16–18).

Regardless of the topic and scope, a successful outbreak exercise requires detailed planning and organization. This begins with recruiting a team and setting up detailed aims and objectives. Thereafter, creating a realistic scenario and planning the conduction are important steps. The length and complexity of these stages depends on the nature and scale of the exercise. Considering the OHEJP SimEx was primarily a discussion-based exercise, the major challenge was to meet the expected needs of eleven countries that had different prerequisites. The scenario had to be generic enough for the exercise to be relevant to each country’s response framework and organizational structure, yet detailed enough to be realistic and capable of resulting in relevant discussions.

To align with the local conditions, the participating countries were given the option to adapt the scenario. Despite this, there were some conflicting opinions from the TA on the exercise content and delivery. Indeed, the variations in *Salmonella* status and relevant regulations and structures amongst the participating countries meant the scenario was more compatible with some countries than others. Further, the main reasons for the lack of reality stated by some participants were the source attribution of a *Salmonella* outbreak to a cattle production unit (usually not regarded as a primary *Salmonella* source), and the inclusion of raw pet food in the exercise. The latter was not yet a relevant market in some of the participating countries, therefore the structures and regulations relating to raw pet food were not well defined. Rather than view this as a disadvantage, the experience for the TA in these countries is uniquely placed to assess the issues and successes each country encountered and provide recommendations for future One Health initiatives. If designing a more detailed exercise, it might be useful to identify countries with a similar prevalence for the selected pathogen and similar relevant systems (e.g., utilizing WGS for surveillance or not), allowing more detailed analysis into specific areas. If countries wish to participate without this alignment, then excluding them from the analysis, or separating countries according to how well they align to the pre-requisites will enable them to benefit from the exercise and provide some important data without affecting the main aims of the exercise. It is important to have a strong representation of all the different OH sectors in the exercise planning team to avoid a biased representation of a specific sector over the others, thus guaranteeing that the exercise can explore relevant topics for each sector.

By including the environmental health sector, future exercises could explore additional aspects of this from a OH perspective often-overlooked sector. When building the scenario, environmental pathogen dissemination was not included as a key event to explore in this exercise, but we noticed that the topic arose during some team discussions and that the absence of environmental health professionals in the TA hindered the development of such discussions. Therefore, the environmental health sector should be considered as an essential part of the holistic OH approach for pathogens that are known to transmit *via* the environment or for novel pathogens where limited knowledge exists and be encompassed in future simulation exercises. Furthermore, in one of the countries unable to secure representation from all three sectors, the TA noted more dissatisfaction regarding the relevance of the scenario, as they could not fully engage in all the injects, an observation that stresses the importance of including all sectors represented in the scenario. Although communication was central to this scenario, an even stronger focus on communication is recommended for future simulation exercises. Indeed, participants identified in some countries the communication staff only had a secondary role during the majority of the OHEJP SimEx. Including from the onset communication experts within the exercise planning team could address this concern. Incorporating more practical tasks (e.g., data sharing exercises) should also be considered to increase the overall participation and engagement.

Based on the weaknesses and strengths identified amongst the different countries it is possible to note common topics requiring improvement to implement the OH strategy to outbreak investigation and management. Countries should strive to set up a OH coordination strategy before a specific need for it is identified, ensuring a well-established organization able to support a prompt and efficient response (1). Interinstitutional guidelines covering relevant authorities and their responsibilities is useful when assembling an interdisciplinary outbreak investigation team, with the relevant authorities working together throughout the different stages of a foodborne outbreak and constructing a suitable joint action plan. During an outbreak, it is important to ensure continuity from beginning to end and maintain collaboration beyond the outbreak investigation, so that the team reviews their strategy and improves it accordingly. Additionally, institutes should implement updated and standardized procedures that can support the outbreak management team, including clarifying the role and responsibilities of each party. All participating countries are in a strong position to understand how far through this process they are and the required steps to achieve the ambitious aim of One-Healthiness. The One Health EJP Joint Integrative Project MATRIX has developed an online tool OH-EpiCap^3^ (25) to facilitate characterizing and improving national One Healthiness through the evaluation of the surveillance system’s capacities and capabilities. Indeed, we would recommend any future OH exercises to encourage the participants to use the OH-EpiCap tool before and after the conduction as one option to quantify the benefits of the exercise.

Establishing a routine of meetings with representatives from the different sectors should be considered a priority for countries aiming to improve their OH strategy. Meeting regularly builds trust and promotes transparency between and within sectors, which is fundamental for a successful cross-sectoral cooperation. To develop an efficient health emergency response system which is capable of quickly adapting to different scenarios, all relevant sectors must be identified and equitably included. Ideally, to enable a swifter decision-making process, at least one member of each sector should have a direct link to governmental bodies to facilitate the communication with policy makers when needed (26, 27). The inclusion of networking and training activities should be considered when planning regular activities to make sure that any updates to the national contingency plans are covered and that new colleagues are included. The action plan should be tested in simulation exercises and reviewed periodically.

The successful implementation of OH structures requires a good understanding of the national and regional context and priorities (27). Implementing actions at local level was overall considered helpful by allowing to better contain the outbreak spread and adapt any needed actions to the regional reality. Nevertheless, OH structures require coordination with central authorities to avoid duplication of resources and efforts.

It was noteworthy that almost all participating institutes declared a dependance on personal and informal communication routes, which compromises the sustainability of interinstitutional communication networks. An effort should be made to implement efficient and official structural communicational channels that can be sustained regardless of personal contacts. These should include contact points at several key organizations, be regularly updated and be easily accessible to all relevant parties while still avoiding complex instructions that diminish compliance.

By centralizing the typing data of pathogen strains from different sources, the data can be made available to the investigation team without delays, helping to move forward with the outbreak investigation. Fragmentation of laboratory services can be time and resource consuming, hinder the harmonization of the results and increase the risk of information delays in the communication between laboratories. When centralization is not feasible or preferred, an effort should be made toward the harmonization of the characterization methods used in the different laboratories so that the results can be transferable and comparable (28). To support the work of reference laboratories, it is important to raise awareness at primary care level to the need of sending isolated strains and epidemiological information to the central laboratories, as well as reinforcing the hospital to laboratory network. In addition, the AH sector should attempt to improve the contact with primary care veterinary services and to establish a stronger network with veterinary practitioners so that isolates from companion animals can be included in national surveillance programmes. Countries needing technical support can reach out to international laboratory networks.

The need for a common data sharing platform that can be used across sectors was a common outcome across the participating countries and its implementation is pivotal in achieving a OH approach. Efficient tools are needed for earlier identification of outbreaks and quicker access to data for analytical studies and source attribution. Ideally, new data sharing platforms should build from and integrate already existing databases, be able to support large amounts of data and allow for multiple users to access simultaneously. To assist in the transition to an integrated strategy, institutes could develop guidelines on interinstitutional data sharing practices. Harmonization efforts could start at an institutional level by promoting a standardized use of internal data management tools by the professionals to avoid the vulnerability of a system dependent on a limited number of people. Ideally, national surveillance systems would be standardized internationally, thereby facilitating a coordinated approach to cross-border foodborne outbreaks.

It was noteworthy that GDPR was highlighted as a major barrier to the implementation of shared data collection across sectors. All personnel with access to the data related to an outbreak will need to be aware of, and comply with, the GDPR that applies to the different data categories and be authorized to work with it. Based on the comments from the participating countries, restricting access to common data sharing platforms to the central authority of each sector was considered preferential. Nevertheless, an efficient communication route needs to be established with the authorities at a regional level to guarantee the quick and efficient implementation of any actions that may be required.

Outbreak investigations comprise of both epidemiological and pathogen-related data. The majority of recommendations and gaps identified were concerning epidemiological data. However, it is important to raise awareness regarding inclusion of pathogen data, in particular genomic data, in national data sharing plans, which are used for cluster identification and source attribution. Ideally, national databases that connect AH, PH and FS laboratories should be created in countries that lack these databases and extended to include more pathogens in countries that already have them in place. To ensure genomic data is used optimally, the protocols used to generate the data and output formats need to be standardized across the different laboratories prior to implementation. As the demand for better and quicker typing techniques increases, there is a need to invest in WGS technologies and building the capacity of skilled teams that can generate and analyze large amounts of genomic data in real time.

Data visualization tools like the FCL, can prove helpful during an outbreak situation by allowing to visualize and analyze complex food networks, help in data collection, tracing back analysis and source attribution. Nevertheless, some points were raised regarding the complicated and time-consuming process of entering data into the FCL platform, and that it may be hard to adapt the tool to a more complex OH incident where the data is too heterogeneous. For the optimal implementation of the tool, it is necessary to improve its interoperability with other information systems and databases (possibly including sequencing data) and offer training on how to use the platform. Countries that showed interest in implementing FCL in their national action plan for foodborne outbreak investigations were given the opportunity to attend a workshop with the tool designers to help with the process. Future multi-country simulation exercises should take the opportunity to include different practical tools such as FCL as it provides a unique platform to fully test the complexities of country specific requirements. Providing a more robust range of suggested improvements benefitting future users.

As noted by many of the countries, the inclusion of communication experts from the different sectors in the outbreak management team is essential to ensure the public perception on the cohesiveness of the team and to promote internal mutual understanding. To assure consistency, their inclusion should precede the assembly of the emergency team and considered in the early construction of OH mechanisms (27). It is important to note that a good communication plan requires flexibility to adapt to rapidly changing situations and should be a dynamic process that involves feedback from both the stakeholders and the communities (27, 29, 30).

The One Health EJP is composed of public institutes in the AH, PH and FS sectors and therefore has close collaboration with national and international stakeholders, including those represented in the OHEJP SimEx Advisory Board. This collaboration has enabled sharing the experiences from OHEJP SimEx to policy makers, whose support is essential for establishing and strengthening the structures needed to implement a OH approach to investigation and management of outbreaks. For the successful implementation of the actions identified here, they need to be assessed taking into consideration each national reality and adapted accordingly. There are several tools and resources available to support decision makers in making the transition to better OH structures and support them in drawing national action plans that can address the major gaps (6).

## 5. Conclusion

The OHEJP SimEx was a successful multi-country national simulation exercise. The results revealed the need for initiatives that can support countries in the practical implementation of OH. With the persistent risk of zoonotic foodborne outbreaks there is a continuous need to invest in prevention and contingency, as well as building capacity to respond to a health emergency, using an OH approach. Future OH simulation exercises can build on the OHEJP SimEx structure and experiences and should try to address the limitations identified. All participants acknowledged the essential tasks to engage with stakeholders and policy makers in order to ensure the framework of practical implementation of a OH approach is supported.

## Data availability statement

The raw data supporting the conclusions of this article will be made available by the authors, without undue reservation.

## Ethics statement

Ethical review and approval was not required for the study on human participants in accordance with the local legislation and institutional requirements. Written informed consent for participation was not required for this study in accordance with the national legislation and the institutional requirements.

## Author contributions

AO and KA conceptualized the project. AB, AO, DM, FA, JB, LT, ML, OP, and RF contributed to scenario design and development, evaluation, and reporting and dissemination of results. AB, AO, DM, FA, JB, LT, ML, OP, PJ, and RF contributed to data collection. OP analyzed the survey data. AO and FA drafted the manuscript. DM contributed to communication, information sharing, and language editing. HI, KA, PJ, and RR contributed to the project design and editing and writing the manuscript. All authors contributed to the article and approved the submitted version.

## Funding

The project has received funding from the European Union’s Horizon 2020 research and innovation programme under Grant Agreement No. 773830.

## Conflict of interest

The authors declare that the OHEJP SimEx project was conducted in the absence of any commercial or financial relationships that could be construed as a potential conflict of interest.

## Publisher’s note

All claims expressed in this article are solely those of the authors and do not necessarily represent those of their affiliated organizations, or those of the publisher, the editors and the reviewers. Any product that may be evaluated in this article, or claim that may be made by its manufacturer, is not guaranteed or endorsed by the publisher.

## Figures and Tables

**Table:** 

AAR	After-Action Review
AH	Animal health
BfR	German Federal Institute for Risk Assessment
ECDC	European Centre for Disease Prevention and Control
EFSA	European Food Safety Authority
EU	European Union
FAO	Food and Agriculture Organization of the United Nations
FCL	FoodChain-Lab
FS	Food safety
GDPR	General Data Protection Regulation
LEs	Local Evaluators
LELs	Local Exercise Leaders
NEL	National Exercise Leader
OH	One Health
OHEJP SimEx	One Health European Joint Programme simulation exercise
One Health EJP	One Health European Joint Programme
PH	Public health
TA	Training Audience
WGS	Whole Genome Sequencing
WHO	World Health Organization
WOAH	World Organisation for Animal Health

## Footnotes

^1^https://onehealthejp.eu/, 2018–2023.

^2^ http://www.medvetnet.org/

^3^ https://freddietafreeth.shinyapps.io/OH-EpiCap/
