# Supplementary material for: A multi-country One Health foodborne outbreak simulation exercise: cross-sectoral cooperation, data sharing and communication
Source: Front Public Health. 2023 Jun 13;11:1121522. doi: 10.3389/fpubh.2023.1121522 (PMC10293640; doi:10.3389/fpubh.2023.1121522)
Supplement: Supplementary file 1 [file Data_Sheet_1.PDF]

Supplementary Material

A multi-country One Health foodborne outbreak simulation exercise: cross-sectoral cooperation, data sharing and communication

OHEJP SimEx Post Exercise Survey

The digital survey will be available for all participants through a link and password. For the survey, the platform EuSurvey was used. The answers provided will be anonymised thus no personal data will be collected by the platform. To be able to share the national results with the National Exercise Leader only information about the country will be collected. The individual results from the survey will not be made public. The compiled results will however be included in SimEx Project's final report and quotes might be used.

Survey questions

Personal information

What was your role during conduction?

A. Local Exercise Leader (LEL)/National Exercise Leader (LEL) B. Training audience C. Local Evaluator D. Other personnel E. Visitor

Which sector do you represent?

A. Animal Health B. Food Safety C. Public Health D. Other

If you were a part of the training audience, what is your profession?

(free text)

General questions regarding preparations and logistics

1. Did you get enough guidance prior to the exercise to understand the purpose and objectives?
2. Did you understand your role in the exercise?

3. What did you think of the exercise planning and organisation?
4. What information or activity could have made you more prepared?

Using a scale from 1-5 (1. Strongly disagree, 2. Disagree, 3. Neutral 4. Agree, 5. Strongly agree) answer the following questions:

1. The venue suited the exercise needs.
2. Available material was adequate.
3. Time provided was sufficient.
4. Time for breaks and lunch was sufficient.
5. The National Exercise Leader (NEL)/Local Exercise Leader (LEL) were well prepared.

Conduction

1. Was the information prior to each part of the exercise delivered in a clear way?
2. How well directed were you in the conduction of the exercise?

For each of the following questions please select an answer (YES/NO) and write a comment elaborating your answer:

1. Did you find the scenario realistic?
2. Did you find the scenario relevant?
3. Whas the pathogen chosen relevant?
4. Do you think the scenario covered all sectors involved (human health, animal health and food safety)?
5. Do you think the scenario was in line with the exercise objectives?
6. Do you think the injects were provided in a manner that mimics a real-life outbreak situation?
7. Was Food Chain Lab Web useful?
8. Did you experience problems when applying FoodChain-Lab Web?
9. Did you miss functionalities in FoodChain-Lab Web?

The objectives

Role and functionality of currently available systems

1. Do you believe this exercise has helped you to be more aware of the currently available warning systems and emergency action plans in place (both at national level and in the European Union) and when they should be activated?

2. Was the exercise successful in stressing the importance of notifying events across sectors?

3. Has your knowledge of the roles and mandates of the other sectors increased? In what way? Will you share this knowledge with your sector in any way?

4. Has your understanding of what other sectors expect from your sector increased?

5. Did you actively provide information about your sector and how you work during the discussions?

6. Do you feel more encouraged to share early warning signals (formal or informal) with the other sectors after this exercise?

Sharing of outbreak data

1. Did you gain an increased understanding of the need to have a harmonized approach for data collation when dealing with a foodborne zoonosis outbreak?

2. Do you think that the exercise revealed any possible flaws of the practices currently applied in your country surveillance programme?

3. Did you identify any gaps or problems when it comes to data sharing? What are those? Are they on a political, technical or institutional level?

4. Does your country or institute use any software or methods to overcome some of these problems, that you wish to inform about?

5. Are problems associated with data sharing something your institute prioritises?

Communication in an outbreak situation

1. Do you think the exercise clarified the role of each sector in an outbreak investigation and the importance of having a coordinated action plan?

2. Did the exercise highlight the advantages of including professionals from all sectors when assembling an outbreak investigation team?

3. Did you gain a better understanding of the different communicational needs and different target audiences?

4. Did the use of Food Chain Lab give you any new insights or better understanding of tracing? In what way?

5. Do you feel encouraged to use a One Health approach and work closer to the other sectors when managing an outbreak in the future?
